# Effect of Emission Current on the Microstructure and Local Mechanical Response of EB-PVD Thermal Barrier Coatings

**DOI:** 10.3390/ma19194056

**Published:** 2026-09-23

**Authors:** Grzegorz Maciaszek, Andrzej Nowotnik

**Affiliations:** Faculty of Mechanical Engineering and Aeronautics, Rzeszów University of Technology, 35-959 Rzeszów, Poland; nowotnik@prz.edu.pl

**Keywords:** thermal barrier coatings, TBCs, electron beam–physical vapour deposition, EB-PVD, ASTM C633, adhesion, indentation, coating hardness

## Abstract

Electron beam physical vapour deposition (EB-PVD) is one of the most advanced technologies for manufacturing thermal barrier coatings (TBCs) with a columnar microstructure and excellent durability under high-temperature service conditions. This study investigates the relationship between electron beam emission current, deposition conditions, microstructure, and mechanical response of 7 wt.% yttria-stabilised zirconia (7YSZ) thermal barrier coatings deposited using an industrial-scale SMART COATER system. The coatings were characterised by microstructural analysis, ASTM C633 tensile testing, and Vickers indentation measurements. Increasing the emission current resulted in higher substrate temperatures, increased evaporation intensity, and a higher deposition rate, leading to thicker coatings with a progressively coarser columnar microstructure. The ASTM C633 testing resulted in cohesive failure within the adhesive layer, indicating that the measured tensile failure stresses provided only a lower bound for coating adhesion. Vickers indentation revealed clear through-thickness hardness variations and higher local hardness for coatings produced under higher-emission-current conditions. Overall, the results demonstrate systematic relationships between deposition conditions, columnar microstructure, and local Vickers hardness of the EB-PVD 7YSZ coatings.

## 1. Introduction

Thermal barrier coatings (TBCs) are advanced multilayer coating systems designed to protect hot-section components of gas turbine engines operating under increasingly demanding thermal conditions [1]. Their application enables an increase in the turbine inlet temperature while simultaneously reducing the temperature experienced by the metallic substrate [2], thus improving engine efficiency, extending the lifetime of the component, and reducing fuel consumption and greenhouse gas emissions [3]. A conventional TBC system consists of a ceramic top coat, typically based on 7–8 wt.% yttria-stabilised zirconia (YSZ), deposited on a metallic bond coat that provides oxidation resistance and promotes adhesion between the ceramic layer and the superalloy substrate [4]. The combination of low thermal conductivity, high melting point, favourable thermal expansion compatibility, and excellent thermal shock resistance has established YSZ as the most widely used ceramic material for industrial thermal barrier coating systems [5].

Among the various deposition technologies, electron beam physical vapour deposition (EB-PVD) is considered one of the most advanced methods for manufacturing high-performance TBC systems [6]. In contrast to plasma-sprayed coatings, EB-PVD coatings exhibit a coarser columnar architecture, characterised by vertically aligned columns separated by intercolumnar gaps [7]. This unique architecture provides excellent strain accommodation during thermal cycling, improved resistance to spallation, and superior durability under severe thermomechanical loading [8]. These advantages have established EB-PVD as the preferred manufacturing technology for turbine blades and vanes used in modern aeroengines [9].

The microstructure developed during the EB-PVD process is highly sensitive to deposition conditions [10]. Process parameters such as substrate temperature, chamber pressure, deposition rate, substrate rotation, source-to-substrate distance, vapour flux and electron beam operating conditions govern the evaporation of the ceramic ingot, the transport of vapour species, and the nucleation and growth of columnar grains [11]. Consequently, even relatively small variations in processing conditions can alter column morphology, porosity, crystallographic texture, coating density, and residual stress state, ultimately affecting the mechanical and thermophysical properties of the deposited coatings [12].

The growth of EB-PVD coatings is governed primarily by the thermal conditions at the substrate surface, where adatom mobility determines the nucleation mechanisms, columnar growth, and the resulting coating morphology [13]. Consequently, substrate temperature is widely recognised as one of the most influential parameters that control the development of EB-PVD coatings [14]. Since substrate temperature governs coating growth during EB-PVD process, any modification of the thermal conditions is expected to affect the resulting microstructure. Changes in column morphology, porosity and coating density consequently influence the local mechanical response of the ceramic layer, including its hardness and adhesion to the substrate [15]. Therefore, correlating substrate temperature with both microstructural evolution and mechanical performance is essential for understanding the mechanisms responsible for coating formation and for optimising industrial EB-PVD processing.

However, the substrate temperature itself is not an independent process variable, but is the result of the combined effect of several operating parameters associated with the electron beam and the deposition system [16]. Among these parameters, the electron beam emission current plays a fundamental role because it determines the power delivered to the evaporated ceramic ingot. Increasing the emission current enhances the evaporation intensity, increases the heat input into the deposition chamber, and consequently modifies the substrate temperature. Nevertheless, the relationship between emission current and substrate temperature is strongly affected by other parameters of the electron beam, including beam-focussing conditions, beam trajectory, and scanning pattern. Therefore, changes in emission current do not necessarily correspond to identical thermal conditions unless the remaining beam parameters are kept constant.

Although numerous studies have investigated the influence of substrate temperature on the microstructure and properties of EB-PVD thermal barrier coatings [17,18,19], comparatively few have addressed how specific electron beam operating parameters determine this temperature under industrial deposition conditions. Moreover, quantitative data correlating electron beam emission current with substrate temperature are rarely reported in the literature, particularly for industrial-scale EB-PVD systems. As a result, the practical relationship between the emission current, thermal conditions during deposition, coating growth, and mechanical performance is not sufficiently understood.

In the present work, the electron beam emission current was intentionally used as the only variable controlling the thermal conditions of the deposition process, while the remaining electron beam parameters, including beam-focussing conditions, were maintained constant. This approach enabled the relationship between emission current, substrate temperature, coating morphology, and local mechanical response to be investigated under industrial EB-PVD conditions. The experiments were carried out using an industrial ALD Smart Coater system, which provides technologically relevant correlations between electron beam operating conditions and coating properties.

## 2. Materials and Methods

Annealed Inconel 718 nickel-based superalloy was used as the substrate material. Two specimen geometries were prepared: cylindrical specimens with dimensions of Ø25.4 mm × 38.1 mm for adhesion testing, according to ASTM C633 [20], and cylindrical specimens with dimensions of Ø25.4 mm × 10 mm for microstructural characterisation.

Before bond coat deposition, all specimens were grit-blasted using corundum particles with a mean size of approximately 60 μm. The abrasive was projected at a pressure of 0.5 MPa from a distance of 100 mm at an angle of 45° to the specimen surface. After grit blasting, the specimens were ultrasonically cleaned in isopropyl alcohol. Subsequently, an aluminide diffusion bond coat was produced by the Above-the-Pack aluminising process. The resulting bond coat exhibited an average thickness of approximately 60 μm and a surface roughness (Ra) of 0.9 to 1.2 μm.

Immediately before ceramic deposition, all specimens were ultrasonically cleaned in isopropyl alcohol and heat-treated in a vacuum furnace at 1050 °C for 30 min. This treatment promoted the formation of a thin thermally grown oxide (TGO) layer, while relieving the residual stresses introduced during the mechanical surface preparation.

The ceramic top coat consisting of 7 wt.% yttria-stabilised zirconia (7YSZ) was deposited by electron beam physical vapour deposition (EB-PVD) using an industrial-scale SMART COATER system (ALD Vacuum Technologies GmbH, Hanau, Germany). The deposition parameters are summarised in Table 1. The electron beam emission current was the only intentionally varied deposition parameter, whereas all remaining electron beam parameters were maintained constant throughout the study.

All EB-PVD processes were performed using 7YSZ ingots supplied by Saint-Gobain Coating Solutions (Avignon, France), with diameters ranging from 62.5 to 62.9 mm. The ingot feed rate was adjusted to ensure stable evaporation conditions for each emission current. During deposition, the specimens were mounted in the holder placed in the central feeder position (Figure 1), symmetrically positioned along the evaporation source, and remained stationary throughout the process. The SMART COATER is equipped with thermocouples integrated into the feeder assembly. The substrate temperatures reported in Table 1 were calculated as the arithmetic mean of the readings from the two thermocouples located immediately adjacent to the central specimen holder, approximately at the mid-height of the specimens. The deposition time was 15 min for all coating variants.

The ceramic coating thickness was determined using a Mitutoyo micrometre (Mitutoyo Corporation, Kanagawa, Japan) by measuring the specimen height before and after deposition. As the specimen bases were masked during the EB-PVD process, coating growth occurred exclusively on the exposed surface. Consequently, the difference between the two measurements corresponded to the thickness of the coating. The reported values represent the arithmetic mean of ten independent measurements performed for each specimen.

The coating microstructure was examined using a Thermo Scientific Phenom XL scanning electron microscope (SEM, Phenom-World B.V., Eindhoven, The Netherlands) on metallographic cross-sections prepared from specimens M1–M3. Quantitative analysis of coating architecture and porosity was performed using digital image processing of cross-sectional SEM micrographs in Fiji (ImageJ version 1.54p) software. Prior to analysis, the spatial scale of each micrograph was calibrated using the corresponding SEM scale bar. For each coating variant, ten representative areas were analysed to account for local microstructural variability, and the resulting values were averaged. Column width and intercolumnar gap width were determined using the linear-intercept method at approximately 75% of the coating thickness measured from the bond coat/top-coat interface. Total porosity was determined by image segmentation. The greyscale threshold was individually adjusted for each micrograph to distinguish the ceramic material from pores and intercolumnar voids, and the total porosity was calculated as the area fraction of the segmented void regions. The results are reported as mean values with corresponding standard deviations.

Adhesion testing was performed in accordance with ASTM C633. The coated specimens (A1–A9) were bonded to uncoated Inconel 718 counterparts of identical dimensions. Before bonding, both the coated specimens and counterparts were ultrasonically cleaned in isopropyl alcohol, while counterpart surfaces were additionally grit blasted using corundum particles (approximately 60 μm) under the same conditions applied during substrate preparation. HTK Ultra Bond 100 adhesive (HTK Hamburg GmbH, Hamburg, Germany) was used for bonding. The adhesive was cured for 90 min at 180 °C under a pressure of 70 N cm^−2^. Tensile adhesion tests were subsequently performed using an Instron 8801 universal testing machine (Figure 2) at a constant crosshead displacement rate of 1 mm/min (Instron, Norwood, MA, USA). The applied load and crosshead displacement were continuously recorded by the testing system. The tensile stress was calculated from the measured load and the nominal cross-sectional area of the bonded cylindrical specimens.

Indentation measurements were performed on polished cross-sections of the coatings using a Micro-Combi Tester (CSM Instruments, Needham, MA, USA) equipped with a Vickers diamond indenter. A maximum load of 500 mN was applied using loading and unloading times of 30 s, corresponding to a loading rate of approximately 16.7 mN s^−1^. A dwell time of 10 s was maintained at the maximum load prior to unloading. Under these conditions, the maximum depth of indentation was approximately 1.5–2.0 μm, corresponding to approximately 1 to 1.5% of the coating thickness.

Indentation measurements were performed in three characteristic regions across the coating thickness: near the bond coat/top coat interface, in the central region of the ceramic coating, and close to the column tips. The centres of the indents were placed approximately 25 μm from the bond coat and 35 μm from the column tips, respectively, while the central measurement position was defined individually for each sample at one-half of the measured coating thickness (Figure 3). These locations were selected to minimise the influence of neighbouring interfaces while ensuring representative evaluation of the local mechanical response of the coating microstructure. The maximum indentation depth was approximately 1.5–2.0 μm, corresponding to only about 1–1.5% of the ceramic coating thickness.

For each coating variant, twenty measurement series were performed. Each series consisted of three indents located in the regions described above, resulting in a total of sixty indents per specimen and twenty measurements for each analysed region. A spacing of 100 μm was maintained between adjacent measurement series to eliminate possible interactions between neighbouring plastic deformation zones and associated crack fields.

The diagonals of the Vickers imprints were determined optically using the Micro-Combi Tester software. To minimise operator-dependent uncertainty, each indentation imprint was measured ten times, and the resulting hardness values were averaged to obtain a single representative Vickers hardness value. Indents exhibiting chipping, coating spallation, or any other defects affecting the accuracy of diagonal determination were excluded from further analysis. At least 16 valid individual measurements were retained in each investigated region and used to calculate the mean hardness and standard deviation.

Because a coating specimen was investigated for each deposition condition, individual indentations were not treated as independent experimental replicates for statistical comparisons between the deposition conditions. The differences in hardness between the coatings produced under different deposition conditions were therefore descriptively evaluated using the mean values and corresponding standard deviations.

The through-thickness variation in Vickers hardness was analysed separately for each coating using the Friedman test. The three measurement locations, in the vicinity of the bond coat, in the central part of the coating, and near the column tips, were treated as repeated measurements within each measurement series. Only series containing valid measurements at all three locations were included in the inferential statistical analysis, resulting in 16, 15, and 12 complete series for samples M1, M2, and M3, respectively. When the Friedman test indicated a significant overall difference, pairwise comparisons between coating depth regions were performed using the Wilcoxon signed rank test with Holm correction for multiple comparisons. Kendall’s coefficient of concordance (W) was reported as a measure of effect size. Statistical significance was assumed at *p* < 0.05.

## 3. Results and Discussion

### 3.1. Effect of Emission Current on Deposition Conditions and Coating Growth

The deposition parameters corresponding to processes I (2.51 A, 0.00936 mbar, 941 °C, 1.4 mm min^−1^, 900 s), II (2.82 A, 0.00931 mbar, 971 °C, 1.5 mm min^−1^, 900 s), and III (3.12 A, 0.00913 mbar, 1029 °C, 1.7 mm min^−1^, 900 s) demonstrate that increasing the electron beam emission current resulted in a progressive increase in substrate temperature and evaporation intensity. Consequently, a higher ingot feed rate was required to maintain stable deposition conditions.

The emission current also plays a key role in shaping the coating thickness. As shown in Table 2, the coating thickness increased systematically with increasing emission current, indicating that higher evaporation rates improved the coating growth rate under otherwise identical processing conditions.

### 3.2. Effect of Substrate Temperature on Coating Microstructure

Representative SEM cross-sections of the coatings deposited under different emission current conditions are presented in Figure 4. All coatings exhibited the characteristic columnar morphology typical of EB-PVD thermal barrier coatings. However, clear differences in coating thickness and column dimensions were observed. The coating deposited at 2.51 A consisted of numerous relatively fine columns, while the coating produced at 3.12 A exhibited a visibly coarser columnar architecture with wider columns.

Quantitative analysis of SEM images confirmed the tendency towards columnar coarsening (Table 3). The mean column width progressively increased from 4.35 ± 1.90 μm for M1 to 4.57 ± 1.98 μm for M2 and 5.03 ± 2.53 μm for M3. In contrast, the mean width of the intercolumnar gap remained nearly unchanged, with values of 0.65 ± 0.19, 0.66 ± 0.19, and 0.63 ± 0.20 μm, respectively. Total porosity did not show a monotonic trend and amounted to 5.12 ± 0.57% for M1, 5.87 ± 0.42% for M2 and 5.03 ± 0.46% for M3. Thus, the observed microstructural evolution was mainly reflected in the gradual increase in column width rather than in systematic changes in intercolumnal gap width or total porosity.

The observed tendency towards columnar coarsening coincided with the increase in substrate temperature accompanying the higher emission current. Elevated substrate temperature is expected to enhance the surface mobility of the arriving species, allowing them to migrate over longer distances before incorporation into the growing coating. This favours competitive columnar growth and may contribute to the development of wider columns, as observed in the present study. In contrast, lower substrate temperatures generally reduce surface mobility and may favour higher nucleation density and the formation of finer columnar features. However, substrate temperature may also affect other characteristics of EB-PVD YSZ coatings, including crystallographic texture and potential phase constitution. Because X-ray diffraction analysis was not performed in the present study, possible changes in these characteristics could not be evaluated and the present discussion is therefore restricted to the morphological evolution directly observed by SEM.

These observations also indicate that the emission current acts primarily as a process-control parameter that modifies the thermal and evaporation conditions during deposition. The associated increase in substrate temperature and coating growth rate coincided with the observed evolution of the columnar architecture. The resulting morphology reflects the balance between vapour deposition kinetics and surface mobility of the arriving species, although additional effects related to crystallographic texture or phase constitution cannot be resolved from the present SEM-based analysis.

### 3.3. Adhesion Behaviour of the EB-PVD Coatings

The results of the tensile adhesion tests are summarised in Table 4. In each sample, the failure occurred cohesively within the adhesive layer rather than at the ceramic/bond coat interface (Figure 5), demonstrating that the adhesion strength of the deposited coatings exceeded the tensile strength of the adhesive.

This failure mode was further confirmed by the tensile stress–strain curves presented in Figure 6, whose characteristic shape is typical of epoxy adhesives.

Because cohesive failure within the adhesive layer occurred in every case, the influence of the emission current on the actual TBC/bond-coat interfacial strength could not be quantified using the ASTM C633 method. However, the results demonstrate that the adhesion strength of the investigated EB-PVD coatings exceeded 67 MPa, while the maximum measured value was greater than 94 MPa. These values are substantially higher than those typically reported for plasma-sprayed thermal barrier coatings, for which adhesion strengths are generally close to 50 MPa [21].

Considering the clear microstructural differences observed between the investigated coatings, it is reasonable to assume that deposition conditions may also influence coating adhesion. However, the absence of commercially available structural adhesives with substantially higher tensile strength currently prevents direct experimental verification using the ASTM C633 methodology. Therefore, future studies should employ a fracture-mechanics-based approach, such as an adapted four-point bending test, capable of promoting controlled crack propagation along the coating interface and allowing evaluation of the interfacial fracture resistance without being limited by cohesive failure of the bonding adhesive [22].

### 3.4. Relationship Between Deposition Conditions and Local Vickers Hardness

Vickers hardness measurements were performed in three characteristic regions of the ceramic top coat, namely in the vicinity of the bond coat, in the central part of the coating, and near the column tips. Indentation tests allowed for characterising the local mechanical response of the 7YSZ EB-PVD coatings produced under different emission current conditions and evaluating the through-thickness hardness distribution. The average Vickers hardness values determined for the three investigated regions are summarised in Table 5 and illustrated in Figure 7.

In the vicinity of the bond coat, the mean hardness systematically increased from 804.6 ± 49.6 HV for coating I to 876.0 ± 26.8 HV for coating II and 923.8 ± 10.3 HV for coating III. A similar trend was observed in the central part of the coating, where the corresponding values were 662.3 ± 28.2, 729.2 ± 23.1, and 759.4 ± 27.7 HV, respectively. Near the column tips, the differences between the coatings were less pronounced, with mean hardness values of 644.6 ± 64.9, 696.5 ± 37.0, and 688.1 ± 42.8 HV. Thus, a systematic increase in hardness was observed with increasing emission current and the associated substrate temperature in the vicinity of the bond coat and in the central part of the investigated coatings, whereas no monotonic trend was observed near the column tips. Since each deposition condition was represented by a single EB-PVD process and one coating specimen, these differences are interpreted as trends rather than as statistically confirmed effects of the deposition parameters.

A statistically significant through-thickness variation in Vickers hardness was observed within all three coatings. The Friedman test revealed significant differences between the three investigated coating depth regions for coating I (χ^2^ = 25.125, *p* < 0.001, W = 0.785), coating II (χ^2^ = 28.133, *p* < 0.001, W = 0.938), and coating III (χ^2^ = 24.000, *p* < 0.001, W = 1.000). Post-hoc Wilcoxon signed-rank tests with Holm correction showed that, for coating I, the hardness in the vicinity of the bond coat was significantly higher than that in the central part of the coating and near the column tips (*p* < 0.001), whereas no significant differences were observed between the central part and the region near the column tips (*p* = 0.404). For coatings II and III, significant differences were observed between all three coating-depth regions (*p* ≤ 0.002).

In all three investigated coatings, the highest mean hardness was measured in the vicinity of the bond coat and decreased toward the outer part of the ceramic layer, demonstrating a pronounced through-thickness hardness gradient. The decrease from the region in the vicinity of the bond coat to the central part of the coating was similar for all three coatings, amounting to approximately 17–18%, while the decrease towards the column tips ranged from approximately 20 to 26%. This behaviour is consistent with the evolution of the columnar microstructure during EB-PVD coating growth and with previous observations reported in the literature [23].

The higher hardness values observed for coatings II and III in the vicinity of the bond coat and in the central part of the coating coincided with the increase in the emission current, the associated increase in the substrate temperature, and the microstructural changes described above. However, these relationships should not be interpreted as evidence of a direct causal effect of emission current or substrate temperature on hardness. Similarly, observed columnar coarsening should not be considered the sole factor governing the local mechanical response, as other microstructural characteristics, including porosity, intercolumnar defects, crystallographic texture, and residual stresses, can also contribute to the measured hardness. In particular, possible changes in crystallographic texture or phase constitution cannot be excluded; however, these effects could not be assessed in the present study because XRD measurements were not included in the experimental program. Therefore, the present results indicate a systematic relationship between the deposition conditions, microstructural evolution, and local Vickers hardness rather than an independently quantified effect of a single process parameter.

## 4. Conclusions

The present study demonstrated that the electron beam emission current can serve as an effective process-control parameter for modifying the growth conditions of EB-PVD thermal barrier coatings. Increasing the emission current was accompanied by an increase in substrate temperature and evaporation intensity, resulting in increased coating thickness and the development of a coarser columnar microstructure.

The ASTM C633 tensile tests resulted exclusively in cohesive failure within the adhesive layer rather than at the TBC/bond-coat interface. Consequently, the intrinsic interfacial adhesion strength of the investigated coatings could not be determined. The recorded tensile failure stresses ranged from 67.6 to 94.2 MPa and therefore provide a lower bound for the adhesion strength of the investigated coating systems under the applied testing conditions.

Indentation measurements revealed a systematic increase in mean Vickers hardness from coating I to coating III in the vicinity of the bond coat and in the central part of the ceramic layer, coinciding with the increasing emission current and the associated substrate temperature. No monotonic trend was observed near the column tips. Since each deposition condition was represented by a single EB-PVD process and one coating specimen, these differences should be regarded as trends observed for the investigated coatings rather than as statistically confirmed effects of the deposition parameters. However, statistical analysis confirmed a significant through-thickness variation in Vickers hardness within each of the three coatings, with the highest mean hardness consistently observed in the vicinity of the bond coat.

The observed hardness variations coincided with the evolution of the columnar microstructure; however, they should not be attributed solely to columnar coarsening. Other microstructural characteristics, including porosity, intercolumnar defects, crystallographic texture, and residual stresses, may also contribute to the local mechanical response of EB-PVD 7YSZ coatings. Therefore, the results indicate a systematic relationship between the applied deposition conditions, microstructural evolution, and local Vickers hardness, rather than an independently quantified effect of a single process parameter.

From a practical perspective, the present work establishes a direct process–structure–property relationship for an industrial EB-PVD system. The presented approach provides a practical framework for controlling substrate temperature through adjustment of the emission current, thereby enabling modification of the coating microstructure and mechanical performance. This helps improve understanding of the EB-PVD process and offers valuable guidance for the industrial manufacturing of advanced thermal barrier coatings. A limitation of the present study is the absence of XRD analysis; consequently, possible changes in phase constitution and crystallographic texture associated with the investigated deposition conditions could not be determined and should be addressed in future work.

## Figures and Tables

**Figure 1:**
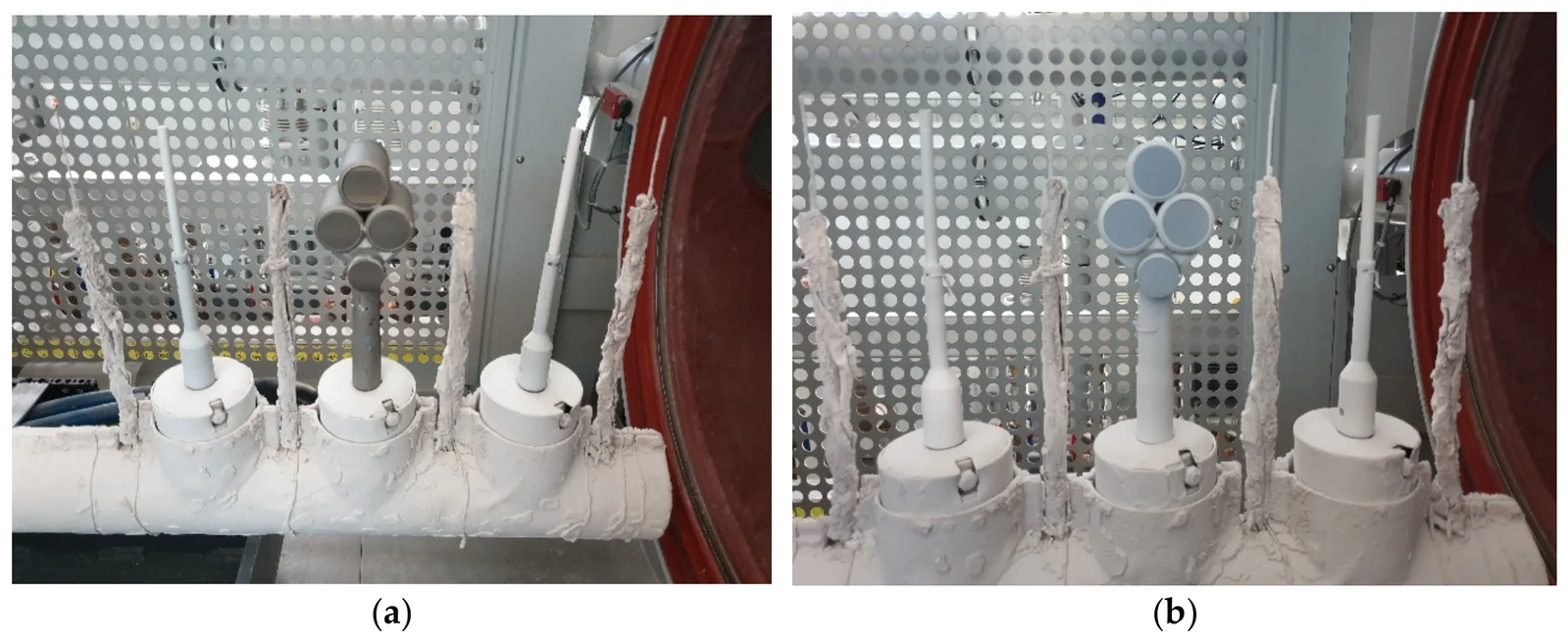
Specimen holder mounted in the central section of the feeder: (**a**) before the EB-PVD process; (**b**) after the EB-PVD process.

**Figure 2:**
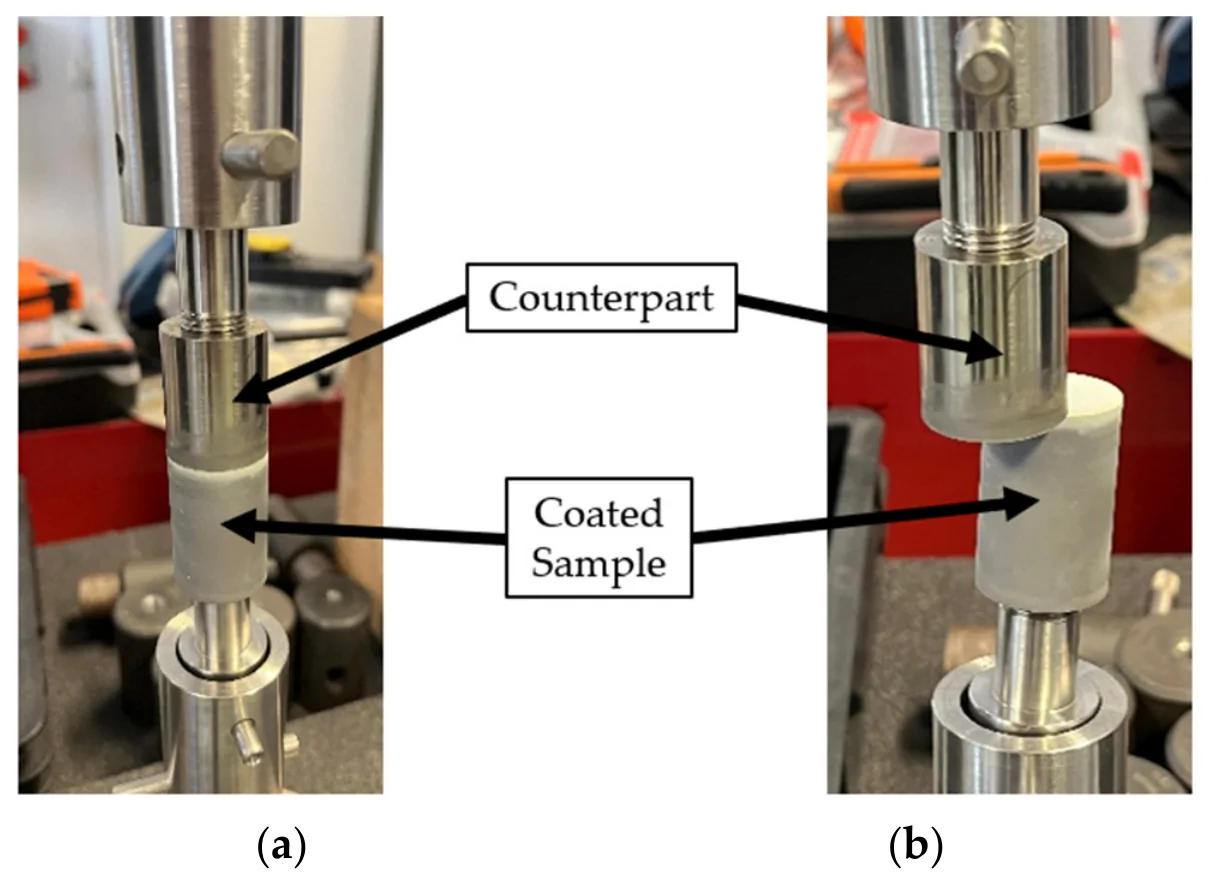
The coated sample glued to counterpart: (**a**) before the tensile adhesion test; (**b**) after the tensile adhesion test.

**Figure 3:**
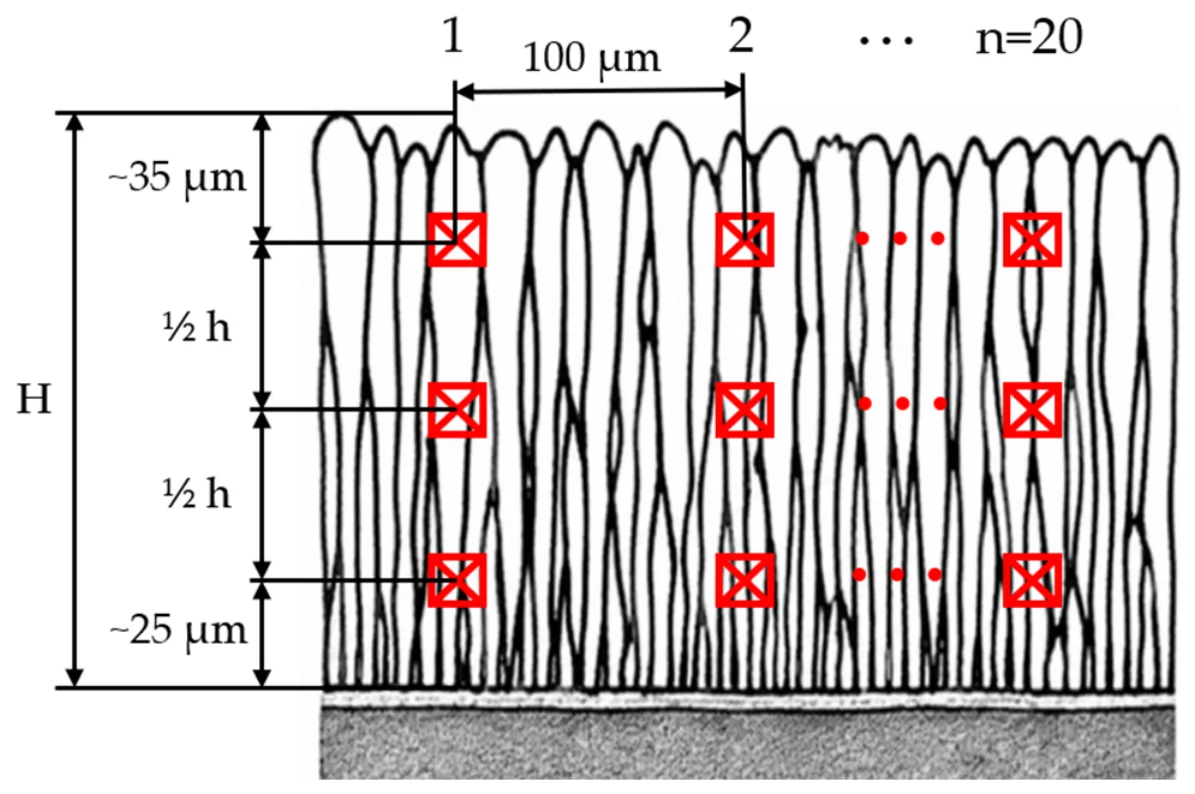
Schematic representation of the TBC cross-section indicating the locations selected for indentation measurements. H denotes the thickness of the coating.

**Figure 4:**
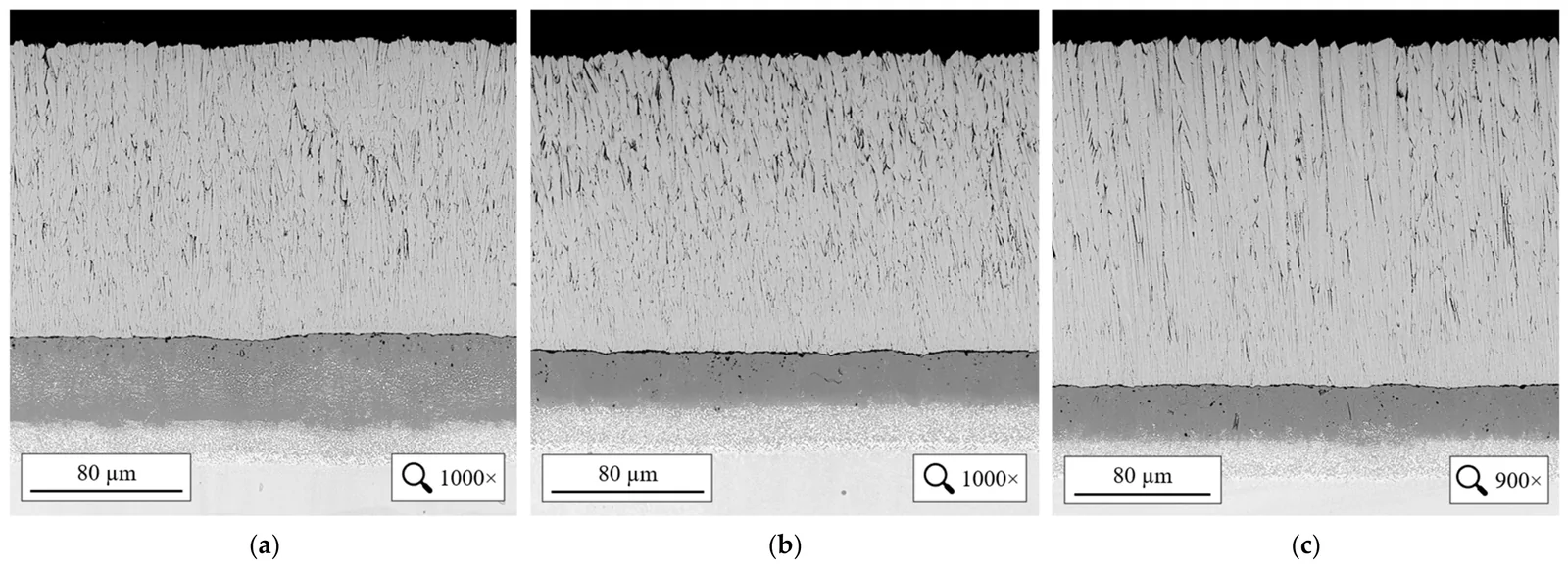
SEM cross-sectional morphologies of 7YSZ coatings deposited using electron beam emission currents of: (**a**) 2.51 A (specimen M1), (**b**) 2.82 A (M2), and (**c**) 3.12 A (M3).

**Figure 5:**
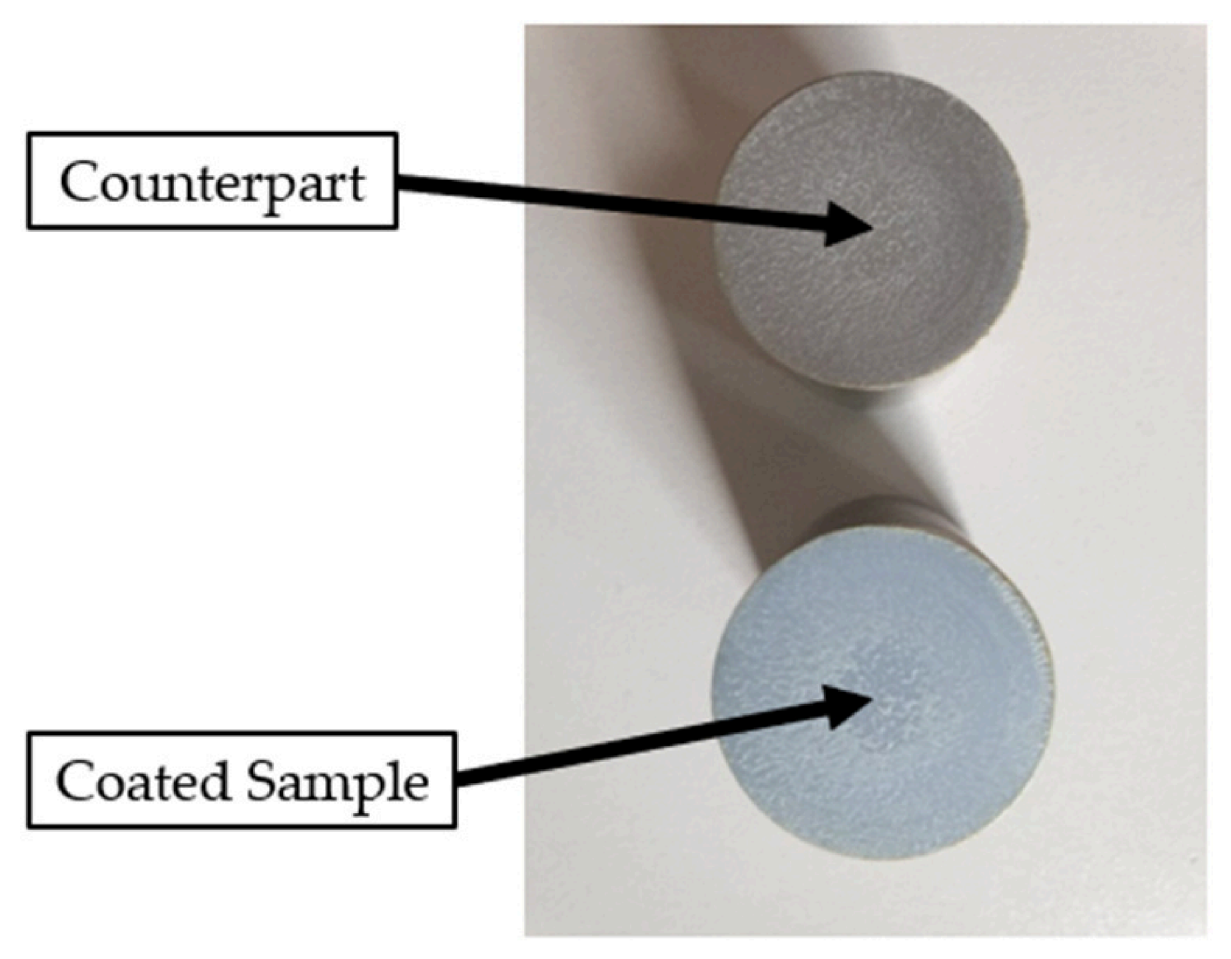
Representative specimen and counterpart after tensile testing showing cohesive failure within the adhesive.

**Figure 6:**
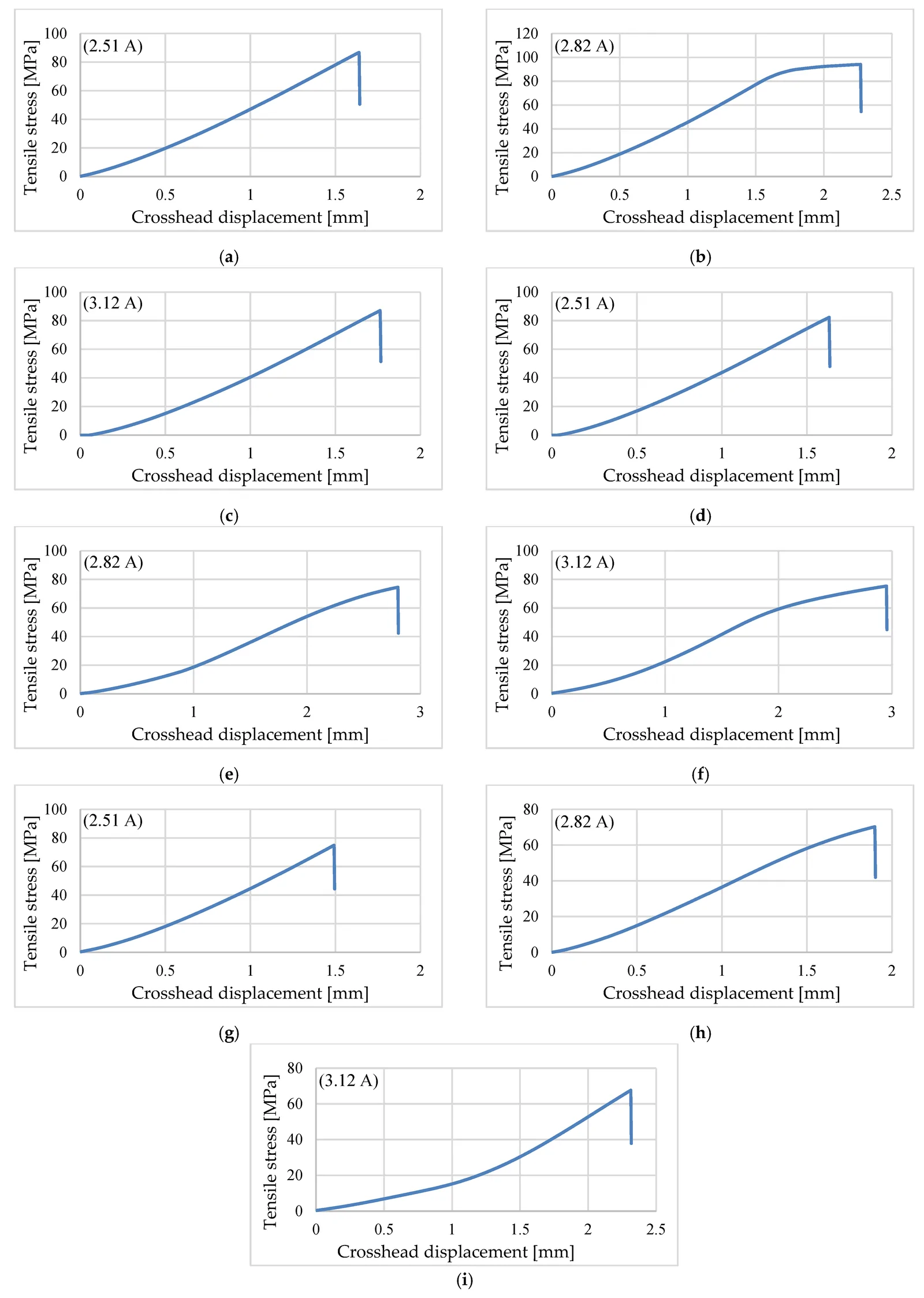
Tensile stress–displacement curves obtained during adhesion testing of bonded specimen: (**a**) A1 (86.8 MPa); (**b**) A4 (94.2 MPa); (**c**) A7 (87.0 MPa); (**d**) A2 (82.3 MPa); (**e**) A5 (74.6 MPa); (**f**) A8 (75.4 MPa); (**g**) A3 (74.9 MPa); (**h**) A6 (70.3 MPa); (**i**) A9 (67.6 MPa).

**Figure 7:**
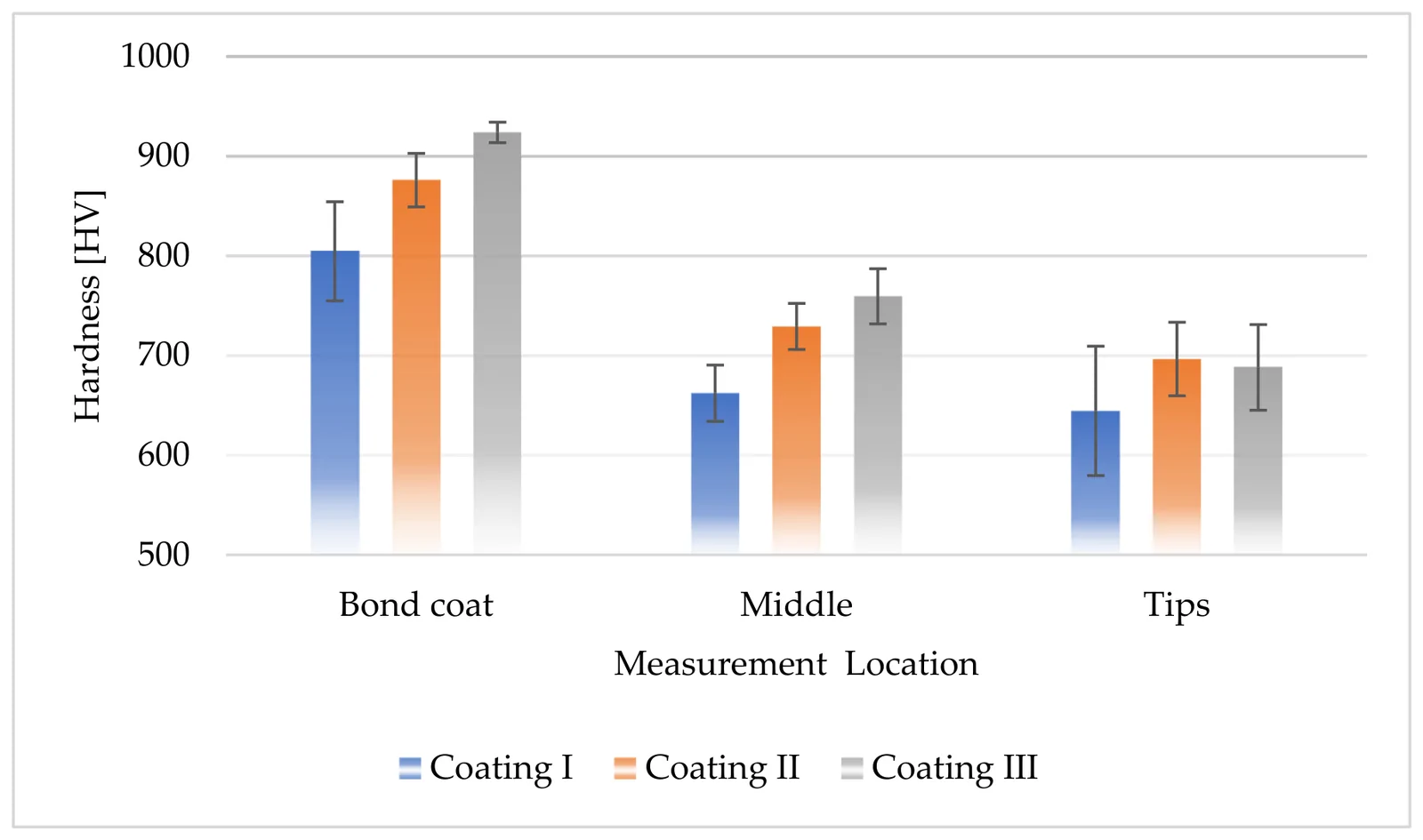
Comparison of average Vickers hardness values measured in the three investigated regions of the coatings. Error bars represent standard deviations.

**Table 1:** EB-PVD process parameters.

Trial Number	Samples Coated	Emission Current [A]	Pressure [mbar]	Temperature [°C]	Feed Rate [mm/min]	Duration [s]
I	A1, A2, A3, M1	2.51	0.00936	941	1.4	900
II	A4, A5, A6, M2	2.82	0.00931	971	1.5	900
III	A7, A8, A9, M3	3.12	0.00913	1029	1.7	900

**Table 2:** Effect of electron beam emission current on coating thickness.

EB-PVD Trial Number	Emission Current [A]	Specimen	Average Coating Thickness [µm]
I	2.51	A1	155
		A2	154
		A3	154
		M1	156
II	2.82	A4	172
		A5	169
		A6	172
		M2	168
III	3.12	A7	205
		A8	203
		A9	204
		M3	203

**Table 3:** Quantitative architectural parameters and porosity of EB-PVD 7YSZ coatings.

Specimen	Emission Current [A]	Column Width [μm]	Intercolumnar Gap Width [µm]	Total Porosity [%]
M1	2.51	4.35 ± 1.90	0.65 ± 0.19	5.12 ± 0.57
M2	2.82	4.57 ± 1.98	0.66 ± 0.19	5.87 ± 0.42
M3	3.12	5.03 ± 2.53	0.63 ± 0.20	5.03 ± 0.46

**Table 4:** Results of ASTM C633 tensile adhesion tests.

Specimen	Emission Current [A]	Maximum Load [N]	Tensile Strength [MPa]
A1	2.51	43976	86.8
A4	2.82	47713	94.2
A7	3.12	44107	87.0
A2	2.51	41715	82.3
A5	2.82	37795	74.6
A8	3.12	38202	75.4
A3	2.51	37960	74.9
A6	2.82	35626	70.3
A9	3.12	34273	67.6

**Table 5:** Average Vickers hardness values measured in the investigated coating regions.

Measurement Location	Coating I (2.51 A) Hardness [HV]	Coating II (2.82 A) Hardness [HV]	Coating III (3.12 A) Hardness [HV]
Near the Bond Coat	804.6 ± 49.6	876.0 ± 26.8	923.8 ± 10.3
In the Middle	662.3 ± 28.2	729.2 ± 23.1	759.4 ± 27.7
Close to the Tips	644.6 ± 64.9	696.5 ± 37.0	688.1 ± 42.8

## Data Availability

The original contributions presented in this study are included in the article. Further inquiries can be directed to the corresponding author.

## Abbreviations

The following abbreviations are used in this manuscript:

TBC	Thermal Barrier Coating
EB–PVD	Electron Beam Physical Vapour Deposition
7YSZ	7 wt.% Yttria-Stabilised Zirconia
SEM	Scanning Electron Microscopy
HV	Vickers hardness
